# High expression of serine and arginine-rich splicing factor 9 (*SRSF9*) is associated with hepatocellular carcinoma progression and a poor prognosis

**DOI:** 10.1186/s12920-022-01316-7

**Published:** 2022-08-15

**Authors:** Guoshun Zhang, Bin Liu, Hua Shang, Guikai Wu, Diyang Wu, Liuqing Wang, Shengnan Li, Zhiyuan Wang, Suying Wang, Juxiang Yuan

**Affiliations:** 1 School of Public Health, North China University of Science and Technology, Tangshan, 063210 Hebei Province People’s Republic of China; 2 Department of Gastroenterology, Affiliated Hospital of North China University of Technology, Tangshan, 063007 Hebei Province People’s Republic of China; 3 Department of Gastroenterology, Chaisang District People’s Hospital, Jiujiang, 332199 Jiangxi Province People’s Republic of China; 4 Blood Purification Department of Tangshan Infectious Disease Hospital, Tangshan, 063026 Hebei Province People’s Republic of China; 5 Department of Gastroenterology, Tangshan Workers’ Hospital, Tangshan, 063003 Hebei Province People’s Republic of China; 6 Department of Gastroenterology, Hongci Hospital, Tangshan, 063009 Hebei Province People’s Republic of China

**Keywords:** *SRSF9*, Hepatocellular carcinoma, Biomarkers, cg06116271, Prognosis

## Abstract

**Background:**

Serine and arginine-rich splicing factor 9 (*SRSF9)* has been linked to the occurrence and progression of various cancers; however, its effects and mechanism of action hepatocellular carcinoma (HCC) have not been reported. In this study, we used a bioinformatics approach and in vitro assays to evaluate the expression of *SRSF9* in HCC, its prognostic value, and its underlying regulatory mechanisms, including analyses of related pathways and the role of methylation.

**Methods:**

Transcriptomic and DNA methylation data for 357 HCC cases and 50 paratumor tissues in The Cancer Genome Atlas database were obtained. Additionally, protein expression data for cell lines and tissue samples were obtained from the Human Protein Atlas. The CMap databased was used to predict candidate drugs targeting *SRSF9.* Various cell lines were used for in vitro validation.

**Results:**

*SRSF9* expression was significantly elevated in HCC and was negatively regulated by its methylation site cg06116271. The low expression of *SRSF9* and hypermethylation of cg06116271 were both associated with a longer overall survival time. A correlation analysis revealed ten genes that were co-expressed with *SRSF9*; levels of *CDK4*, *RAN*, *DENR*, *RNF34,* and *ANAPC5* were positively correlated and levels of *RBP4*, *APOC1*, *MASP2*, *HP,* and *HPX* were negatively correlated with *SRSF9* expression. The knockdown of *SRSF9 *in vitro inhibited the proliferation and migration of HCC cells and significantly reduced the expression of proteins in the Wnt signaling pathway (DVL2 and β-catenin) and cell cycle pathway (Cyclin D and Cyclin E). A CMap analysis identified two drugs, camptothecin and apigenin, able to target and inhibit the expression of *SRSF9*.

**Conclusions:**

This study expands our understanding of the molecular biological functions of *SRSF9* and cg06116271 and provides candidate diagnostic and therapeutic targets for HCC.

**Supplementary Information:**

The online version contains supplementary material available at 10.1186/s12920-022-01316-7.

## Highlights

*SRSF9* is a potential independent risk factor for HCC.

High methylation status of cg06116271 predicted a better prognosis in HCC.

*SRSF9* knockdown inhibited the Wnt signaling pathway and cell cycle pathway.

*SRSF9* may serve as a potential therapeutic target for HCC.

**Supplementary Information:**

The online version contains supplementary material available at 10.1186/s12920-022-01316-7.

## Introduction

Hepatocellular carcinoma (HCC) is the most common primary malignant tumor. Due to the high heterogeneity of tumor cells and high malignancy, the prognosis is ultimately poor [1]. To improve the prognosis of patients with HCC, comprehensive treatment strategies based on surgery have been adopted. With the application of novel treatment schemes, including immunotherapy [2], the survival time still has not increased significantly. The complex mechanisms and multiple risk factors for HCC drive research aimed at revealing the pathogenesis of HCC and the development of corresponding treatment strategies. At the molecular level, HCC is characterized by expression changes in a large number of genes, abnormal epigenetic regulation, and changes in the activity of cellular pathways, leading to abnormalities in the microenvironment of HCC as well as proliferation, differentiation, migration, and even metastasis [3]. Therefore, identifying genes related to prognosis may provide a basis for the development of targeted biotherapies to improve the prognosis of patients with HCC.

Serine and arginine-rich splicing factor 9 (*SRSF9*) is an important gene with regulatory effects in the pathological process of a variety of tumors. For example, an abnormal increase in the expression level of *SRSF9* in oral squamous cell carcinoma leads to a significant reduction in survival time via the regulation of alternative splicing [4]. However, there are no comprehensive and systematic reports on the role of *SRSF9* in HCC. Therefore, we collected transcriptomic and DNA methylation data for a large sample of patients with HCC along with detailed clinical information from The Cancer Genome Atlas (TCGA) database to explore associations between *SRSF9* and prognosis, clinical characteristics, and genetic pathways in HCC. We verified the results of the bioinformatics analyses by knocking down the expression of *SRSF9 *in vitro. This is the first detailed study of the role of *SRSF9* in the pathological process of HCC. This study not only expands our understanding of the roles of *SRSF9* in tumor biology but also clarifies the pathogenesis of HCC at the molecular level. Our results provide sufficient evidence for the clinical value of *SRSF9* as a biological target for the treatment of HCC.

## Material and methods

### Data collection

TCGA was jointly launched by the National Cancer Institute (NCI) and National Human Genome Research Institute (NHGRI) in 2006 [5]. It is an important data source for global cancer research, including clinical data, mRNA expression data, and methylation data for various cancers. Data for 374 HCC samples and 50 paratumor tissues were used for a comparative analysis of *SRSF9* expression. Samples with missing information, such as survival time and tumor grade, were screened. Finally, 357 HCC samples with complete clinical and methylation data were retained for further data mining and analyses (Additional file 1: Table S1).

The Human Protein Atlas (HPA) (www.proteinatlas.org) is one of the most widely accessed protein databases in the world, including continuously updated tissue and cell distribution information for numerous human proteins [6]. The expression levels of proteins in 64 cell lines, 48 normal human tissues, and 20 tumor tissues detected by immunoassays are available in this database of the human proteome. We utilized the HPA database to query the expression levels of the protein encoded by *SRSF9* in normal liver tissues and HCC tissues. To validate the results of the bioinformatics analysis, five pairs of matched HCC and normal liver samples from laboratory were used to verify the protein levels of *SRSF9* at the tissue level.

### Gene set enrichment analysis

A gene set enrichment analysis (GSEA) is a conventional tool for obtaining biological information from gene expression data [7], which is based on the KEGG pathway database [8]. The enrichment of genes was analyzed by an initial database of defined gene sets. Functional enrichment of genes showing correlated expression patterns with *SRSF9* in HCC-related pathways were performed using GSEA 3.0.jar. *P* < 0.05 and false discovery rate (FDR) < 0.25 were thresholds for significance.

### CMap analysis

The Connectivity Map (CMap) database contains transcriptome data for cultured cells treated with active small molecules and uses a pattern matching algorithm to predict gene expression changes caused by drugs. In this study, genes with positive co-expression were regarded as upregulated and vice versa. Then, a CMap analysis was performed to obtain a collection of drug molecules. The top two drugs based on the strength of the negative correlation were selected as candidate drugs for the treatment of HCC.

### Cell culture and treatment

The normal liver cell line L02, hepatoblastoma cell line HepG2, and hepatocellular carcinoma cell lines Huh-7 and Hep3B were purchased from the American Type Culture Collection (ATCC, Manassas, VA, USA). Cells were cultured in DMEM, supplemented with 10% fetal bovine serum (FBS) and 1% penicillin, and placed in a sterile incubator with 5% CO_2_ at 37 °C. In the drug intervention experiments, cells were treated with *S*-adenosyl methionine (SAM) for 8 h, decitabine (DAC) for 72 h, camptothecin for 12 h, and apigenin for 12 h. In the *SRSF9* knockdown experiment, control shRNA (shNC) or shRNA targeting *SRSF9* (shSRSF9) (5′-GATCCGGAAGGATCACATGCGAGAATTCAAGAGATTCTCGCATGTGATCCTTCTTTTTA-3′) was transfected into Huh-7 and Hep3B cells using Lipo2000 (Invitrogen, Waltham, MA, USA). After 24 h of incubation, the cells were used in subsequent experiments.

### Real-time RT-PCR

Total RNAs from cells and tissues were extracted using TRIzol reagent (Invitrogen) and then reverse transcribed into cDNA using the Titan One Tube RT-PCR Kit (Roche, Mannheim, Germany). The expression of selected genes was detected by RT-PCR via TaqMan Fast Advanced Master Mix (Thermo Scientific, Waltham, MA, USA). *GAPDH* was recognized as an internal reference, and the primer sequences were as follows: 5′-CTACAAGTACGGCCGCATCC-3′ (sense) and 5′-CCCCGACCTCCATAAGTCCT-3′ (antisense) for *SRSF9*; 5′-CACCCACTCCTCCACCTTTGA-3′ (sense) and 5′-ACCACCCTGTTGCTGTAGCCA-3′ (antisense) for *GAPDH*. All experiments were repeated three times, and *P*-values less than 0.05 were statistically significant.

### Immunoblotting and immunofluorescence

Whole proteins of tissues and treated cells were extracted using radioimmunoprecipitation (RIPA) lysis buffer containing a protease inhibitor cocktail (Proteintech, Wuhan, China). Centrifugation was performed at 4 °C and 13,000 rpm for 20 min. The protein samples were analyzed by 10% SDS-PAGE and transferred to a PVDF membrane (Thermo Scientific). The membrane was sealed with 5% skim milk at 24 °C for 1 h, incubated with primary antibodies (SFRS9, Cat 17926-1-AP, Proteintech; DVL2, Cat 12037-1-AP, Proteintech; β-catenin, Cat 17565-1-AP, Proteintech; Cyclin D, Cat 26939-1-AP, Proteintech; Cyclin E, Cat 11554-1-AP, Proteintech) at 4 °C overnight, and washed with TBST three times (10 min each). Furthermore, the membrane was incubated with HRP-conjugated secondary antibody (HRP, Cat SA00001-2; Proteintech) at room temperature for 1 h, and washed with TBST three times (10 min each). Finally, the HRP signal was detected using the chemiluminescence detection system (Applygen Technologies, Beijing, China).

For immunofluorescence experiments, adherent cells were fixed with 4% paraformaldehyde and then 0.3% Triton X-100 was applied to increase the permeability of the cell membrane. Cell samples were sealed in 10% serum at room temperature for 1 h and then incubated with the specific antibody (Ki67, Cat 27309-1-AP; Proteintech) overnight at 4 °C. Samples were washed with 0.5% TBST and incubated with the secondary antibody labeled with fluorescent (Alexa Fluor 594, Cat A11037; Invitrogen) for 1 h at room temperature in the dark. Then, samples were incubated with DAPI solution for 10 min. Finally, images were obtained using a fluorescent microscope and processed using ImageJ.

### MTT assay

Huh-7 and Hep3B cells treated with shRNA for 24 h were seeded into 96-well plates at a density of 2000 cells per well. Then, 20 μL of MTT solution (5 mg/mL) was added to each well at 0 h, 24 h, 48 h, 72 h, and 96 h and then incubated at 37 °C for 4 h. Formazan crystals were dissolved in 150 μL of DMSO in 96-well plates and incubated in the dark for 15 min. The absorbance of Huh-7 and Hep3B cells at 490 nm was detected by a microplate reader.

### Colony formation and Transwell assays

Huh-7 and Hep3B cells transfected with shRNA for 24 h were resuspended and cultured in a 6-well plate at a density of 500 cells per well. After 14 d of incubation within complete medium, cell colonies were fixed with 4% paraformaldehyde and then stained with crystal violet solution. After images of the staining results were obtained, cell colonies were counted and analyzed.

Huh-7 and Hep3B cells treated with shRNA were resuspended in the medium with 5% FBS. Cells were cultured in Transwell chambers at a density of 10^5^ cells per well. In addition, 600 μL of medium containing 20% FBS was added to the lower chamber of the 24-well plate. After 48 h of incubation, cells that migrated to the outer side of the Transwell membrane were fixed and stained. Stained cells were counted and analyzed.

### Statistical analysis

All experiments were repeated three times independently and all results were analyzed using GraphPad 9.0. The unpaired Student’s *t*-test or one-way ANOVA was used for comparisons between groups. The data are described as means ± standard deviation (SD). *P*-value less than 0.05 was regarded as statistically significant.

## Results

### mRNA and protein expression levels of *SRSF9* were significantly increased in HCC

**Fig. 1:**
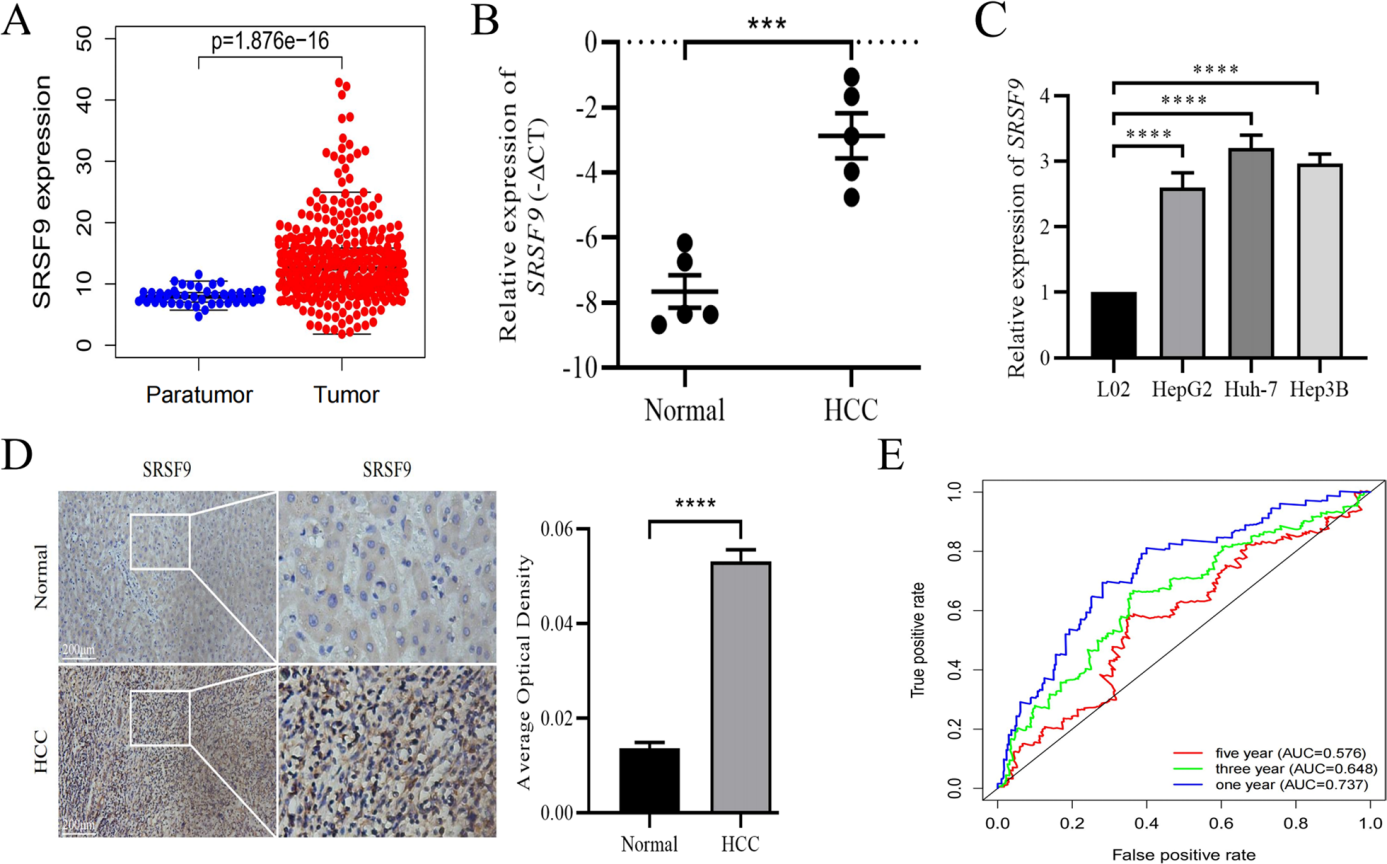
High expression of *SRSF9* in HCC tissues and cell lines. **A** mRNA level of *SRSF9* in 374 HCC tissues and 50 paratumor tissues based on TCGA database; **B** mRNA level of *SRSF9* in HCC and normal tissues from laboratory samples; **C** mRNA level of *SRSF9* in normal liver cells (L02), a hepatoblastoma cell line (HepG2), and HCC cell lines (Huh-7 and Hep3B); **D** IHC results for *SRSF9* in HCC and normal tissues from laboratory samples; **E** Correlation between *SRSF9* expression and 1-year, 3-year, and 5-year survival rates of patients with HCC based on TCGA database. ***P < 0.001, ****P < 0.0001 versus the normal group (or L02 group). AUC > 0.7 indicated a high accuracy. *AUC* area under the curve

In this study, expression changes in *SRSF9* in HCC and its impact on prognosis were evaluated. The mRNA expression of *SRSF9* was significantly higher in HCC tissue samples (n = 374) than in paratumor samples (n = 50 cases) in TCGA (Fig. 1A). To verify these results, we collected HCC and normal tissue samples (n = 5 each) and three HCC cell lines. The RT-qPCR results showed that the mRNA expression of *SRSF9* was significantly higher in both HCC tissue samples and three HCC cell lines than in the control group (Fig. 1B, C). We further examined the protein expression level of *SRSF9* in HCC by immunohistochemistry. *SRSF9* expression was significantly higher in HCC tissues than in control tissues in both the HPA database and clinical samples (Fig. 1D; Additional file 1: Figure S1A). Finally, we found that the expression level of *SRSF9* was correlated with the 1-year survival rate of patients with HCC by an ROC curve analysis based on TCGA data (area under the curve, AUC = 0.737) (Fig. 1E). Based on these results, we speculate that *SRSF9* has an important regulatory role in the pathological process of HCC.

### High expression of *SRSF9* is regulated by DNA methylation

**Fig. 2:**
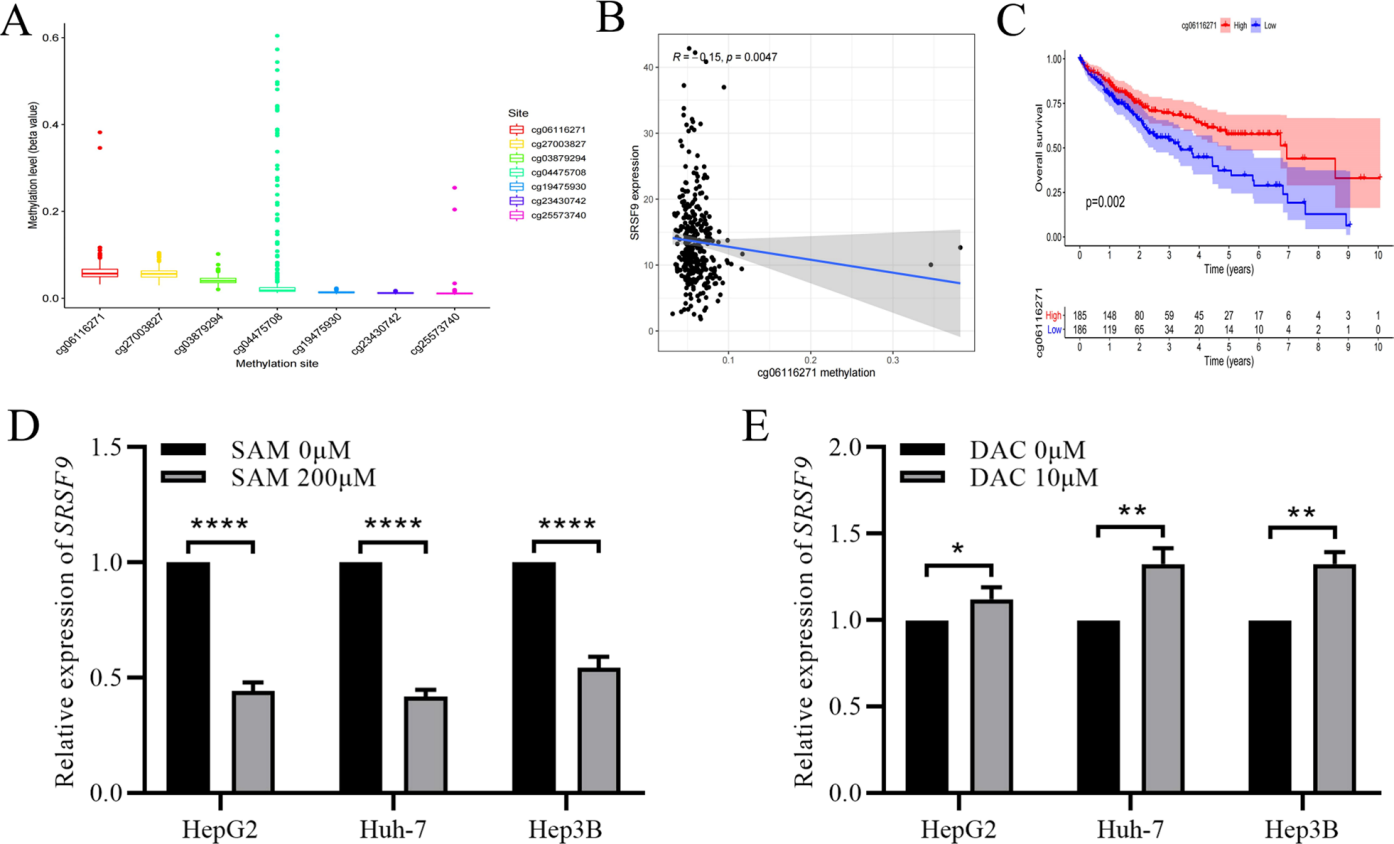
Methylation of *SRSF9.* **A** Methylation status of seven methylation sites of *SRSF9*; **B** Relationship between the methylation level of cg06116271 and the expression of *SRSF9*; **C** Overall survival for patients with high and low methylation status at cg06116271; **D** mRNA level of *SRSF9* in cells treated with SAM; **E** mRNA level of *SRSF9* in cells treated with DAC. *P < 0.05, **P < 0.01, ****P < 0.0001 versus control group. SAM: *S*-Adenosyl methionine; DAC: Decitabine

mRNA expression is frequently regulated by DNA methylation. To explain the high expression of *SRSF9* in HCC, methylation data for HCC were collected from TCGA. Seven methylation sites for *SRSF9* in HCC tissues were identified, and low levels of methylation were maintained at all sites (Fig. 2A). A correlation analysis identified only one methylation site that was significantly correlated with gene expression, i.e., the methylation level of cg06116271 had a negative correlation with *SRSF9* expression (*R* = − 0.15, *P* = 0.0047) (Fig. 2B). A high methylation status of cg06116271, indicating low *SRSF9* expression, was correlated with a better prognosis in HCC (Fig. 2C). These results suggested that methylation contributes to the regulation of the expression of *SRSF9* in HCC. To verify this, 200 μM SAM, which increases DNA methylation levels [9], was used to stimulate selected HCC cell lines. RT-PCR results showed that the mRNA levels of *SRSF9* were remarkably reduced by nearly 50% upon treatment with SAM (Fig. 2D). Conversely, there was a statistically significant upregulation of *SRSF9* expression after treatment with the demethylation drug DAC [10] (Fig. 2E). The results of drug treatment experiments clearly demonstrated that *SRSF9* expression is regulated by DNA methylation; therefore, the high expression of *SRSF9* in HCC can likely be attributed to its hypomethylation status.

### High expression of *SRSF9* is associated with malignant features of HCC

**Fig. 3:**
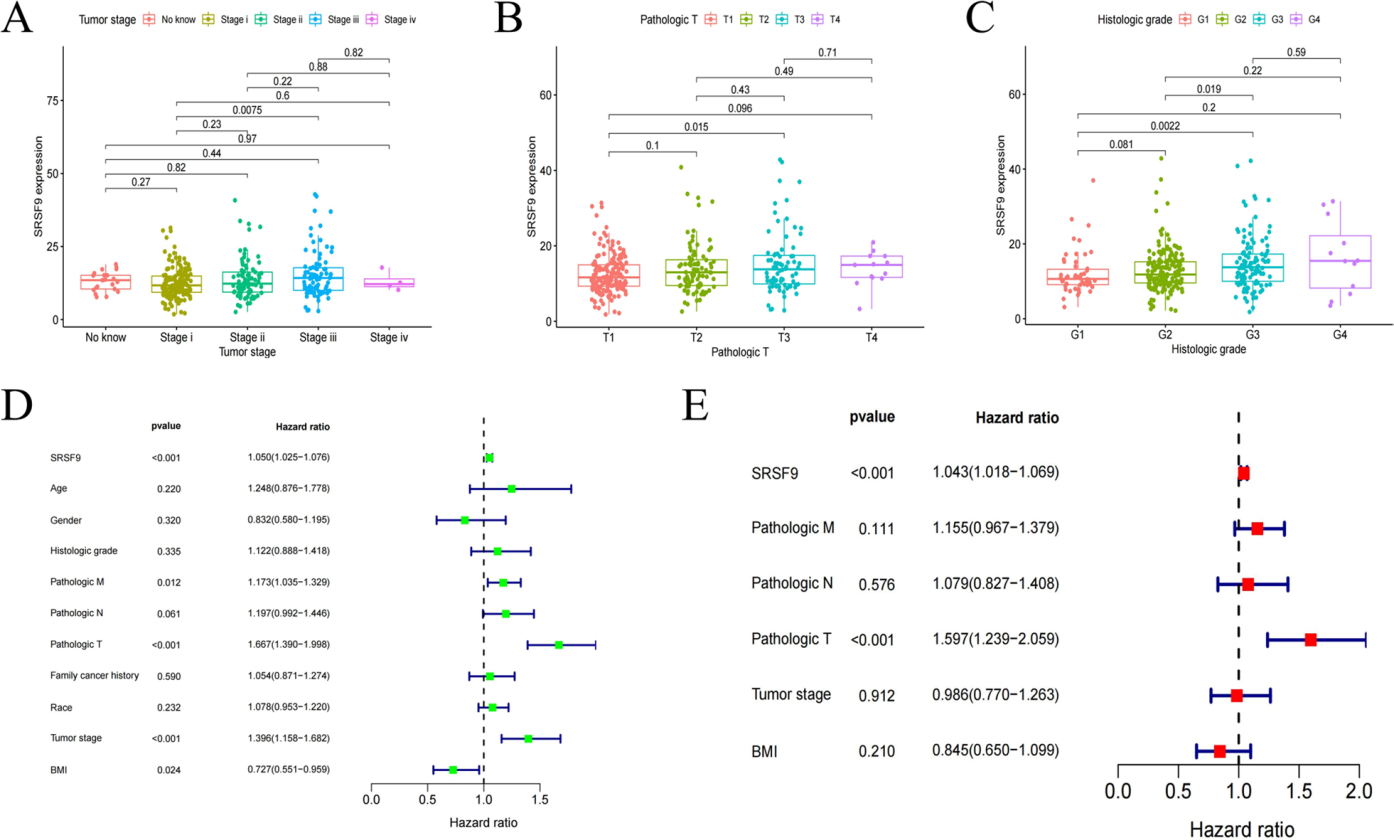
Correlation of *SRSF9* expression with clinical features and prognosis of patients with HCC from TCGA database. **A–C** Relationship between *SRSF9* expression and tumor stage, pathologic T, and histologic grade; **D** Univariate analysis of multiple factors in HCC; **E** Multivariate analysis of various factors contributing to HCC. *P* < 0.05 was considered significant. *BMI* body mass index

Based on the impact of clinical characteristics on prognosis, we evaluated whether *SRSF9* is also related to the poor prognosis of HCC. We selected data for 357 patients with HCC and explored the relationship between *SRSF9* expression and the histopathological stages of HCC by the chi-squared test. As shown in Fig. 3A–C, higher *SRSF9* expression was significantly positively correlated with malignant characteristics of HCC, such as tumor stage III versus stage I (*P* = 0.0075); pathologic T3 versus T1 (*P* = 0.015); histologic grade G3 versus G1 (*P* = 0.0022), and G3 versus G2 (*P* = 0.019). Furthermore, a univariate analysis demonstrated that a variety of factors are unfavorable in HCC, such as *SRSF9*, Pathologic M, Pathologic T, and tumor stage, while BMI is a favorable factor for HCC (Fig. 3D). After excluding other influencing factors, the results of a multivariate analysis indicated that *SRSF9* is an independent prognostic factor for HCC as well as Pathologic T (Fig. 3E). Overall, these results suggested that high *SRSF9* expression acted as an adverse factor associated with HCC.

### High *SRSF9* expression reduces overall survival in HCC by promoting the malignant behavior of tumor cells

**Fig. 4:**
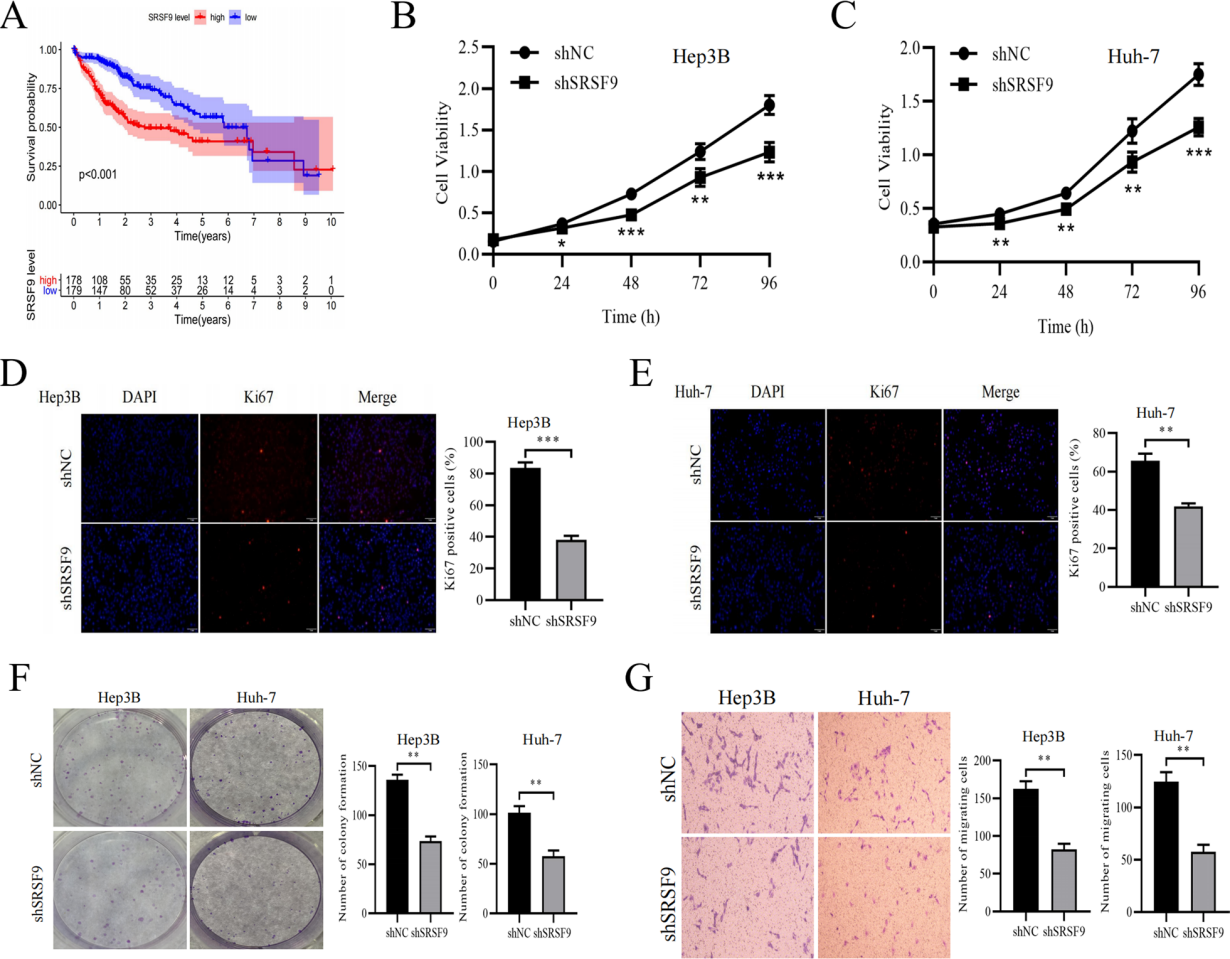
Knockdown of *SRSF9* inhibits the proliferation and migration of HCC cells. **A** Overall survival of patients with HCC with high and low expression of *SRSF9* based on TCGA database; **B–C** Viability of shRNA-treated cells at each assay time point; **D**, **E** Positive staining of Ki67 in different treatment groups; **F** colony staining results for HCC cells treated with shRNA. **G** Staining results for migrated cells from different treatment groups. *P < 0.05, **P < 0.01, ***P < 0.001 versus shNC group

Pathogenic genes often reduce the survival rate of patients with malignant tumors; accordingly, data were divided into high and low expression groups according to the median expression value of *SRSF9*. Then, the Kaplan–Meier (KM) curve showed that overall survival of patients in the high expression group (n = 178) was significantly lower than that of the low expression group (n = 179) (Fig. 1F). In vitro experiments were performed to verify the effect of *SRSF9* on cell behavior in HCC. shRNA was used to knock down the mRNA expression of *SRSF9* in Hep3B and Huh-7 cells (Additional file 1: Figure S1B). Then, a CCK8 assay demonstrated that the rates of proliferation of Hep3B cells and Huh-7 cells with the knockdown of *SRSF9* were significantly lower than those of the control group (Fig. 4B, C). Next, fluorescence staining results for Ki67 (a marker of the proliferative capacity of Hep3B cells and Huh-7 cells) suggested that as the expression level of *SRSF9* decreased, the protein level of Ki67 also decreased (Fig. 4D, E), and the ability of Hep3B cells and Huh-7 cells to form colonies was also inhibited (Fig. 4F). In addition, the migratory ability of shRNA-treated cells was also verified by a Transwell assay, demonstrating that reduced *SRSF9* expression inhibited the migratory ability of Hep3B and Huh-7 cells (Fig. 4G). These results suggest that *SRSF9* plays an important pathogenic role in HCC; however, its mechanism of action needs to be further evaluated.

### *SRSF9* functions via the Wnt signaling pathway and cell cycle related pathways

**Fig. 5:**
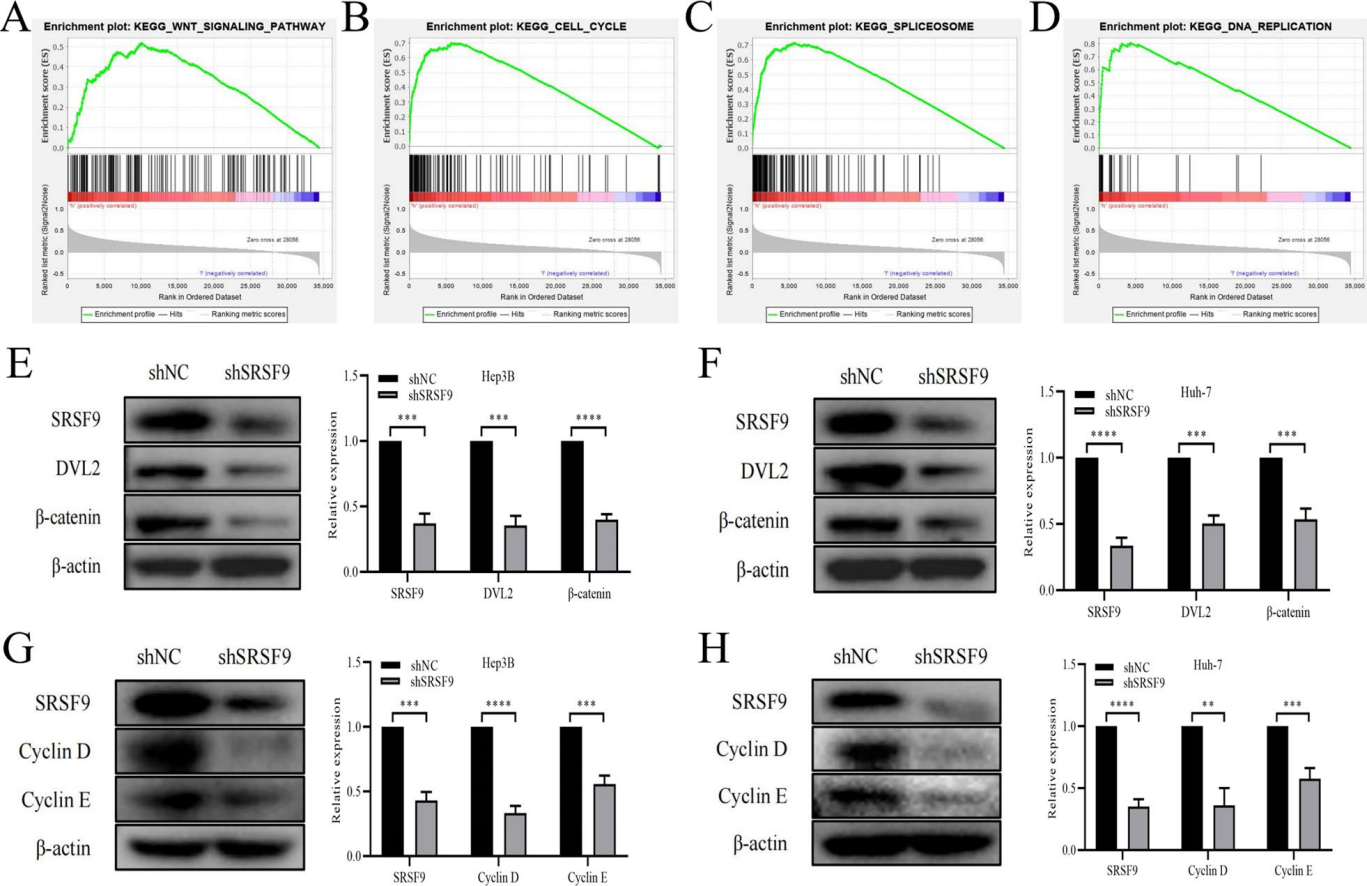
Regulation of the Wnt pathway and cell cycle pathway by *SRSF9*. **A–D** GSEA results indicating that *SRSF9* is involved in the Wnt signaling pathway, cell cycle, spliceosome, and DNA replication; **E**, **F** Expression of proteins in the Wnt signaling pathway in different treatment groups in Hep3B and Huh-7 cells. **G**, **H**, Expression of proteins in the cell cycle pathway in different treatment groups in Hep3B and Huh-7 cells. **P < 0.01, ***P < 0.001, ****P < 0.0001 versus shNC group

A GSEA was performed to predict the signaling pathways mediating the effects of *SRSF9*. The enrichment of *SRSF9* was screened by FDR less than 25%, and related pathways were chosen according to *P* < 0.05. The significant pathways were as follows: Wnt signaling pathway, cell cycle, spliceosome, and DNA replication (Fig. 5A–D). Detailed data are provided in Additional file 1: Table S2. These results suggested that the effects of *SRSF9* are mediated by these signaling pathways, and thus these pathways contribute to the progression of HCC. To further verify the GSEA results, DVL2 and β-catenin, related to the Wnt signaling pathway, were significantly downregulated, as determined by western blotting, in Hep3B cells and Huh-7 cells with *SRSF9* knockdown (Fig. 5E, F). The expression levels of DVL2 and β-catenin were also evaluated in HCC tissues and normal tissues, revealing higher *SRSF9* expression levels in HCC tissues than in normal tissues (Additional file 1: Figure S2A). Furthermore, the cell cycle pathway identified as enriched in the GSEA of *SRSF9* was also validated. The protein expression levels of Cyclin D and Cyclin E, key proteins related to the cell cycle pathway, were also reduced by the knockdown of *SRSF9* in Hep3B and Huh-7 cells (Fig. 5G, H). These results were consistent with those obtained in HCC tissues (Additional file 1: Figure S2B). Collectively, the in vitro experiments confirmed that the function of *SRSF9* may be mediated by various mechanisms, such as regulation of the Wnt signaling pathway and cell cycle pathway, in HCC.

### Co-expressed genes and potential therapeutic drugs targeting *SRSF9*

**Fig. 6:**
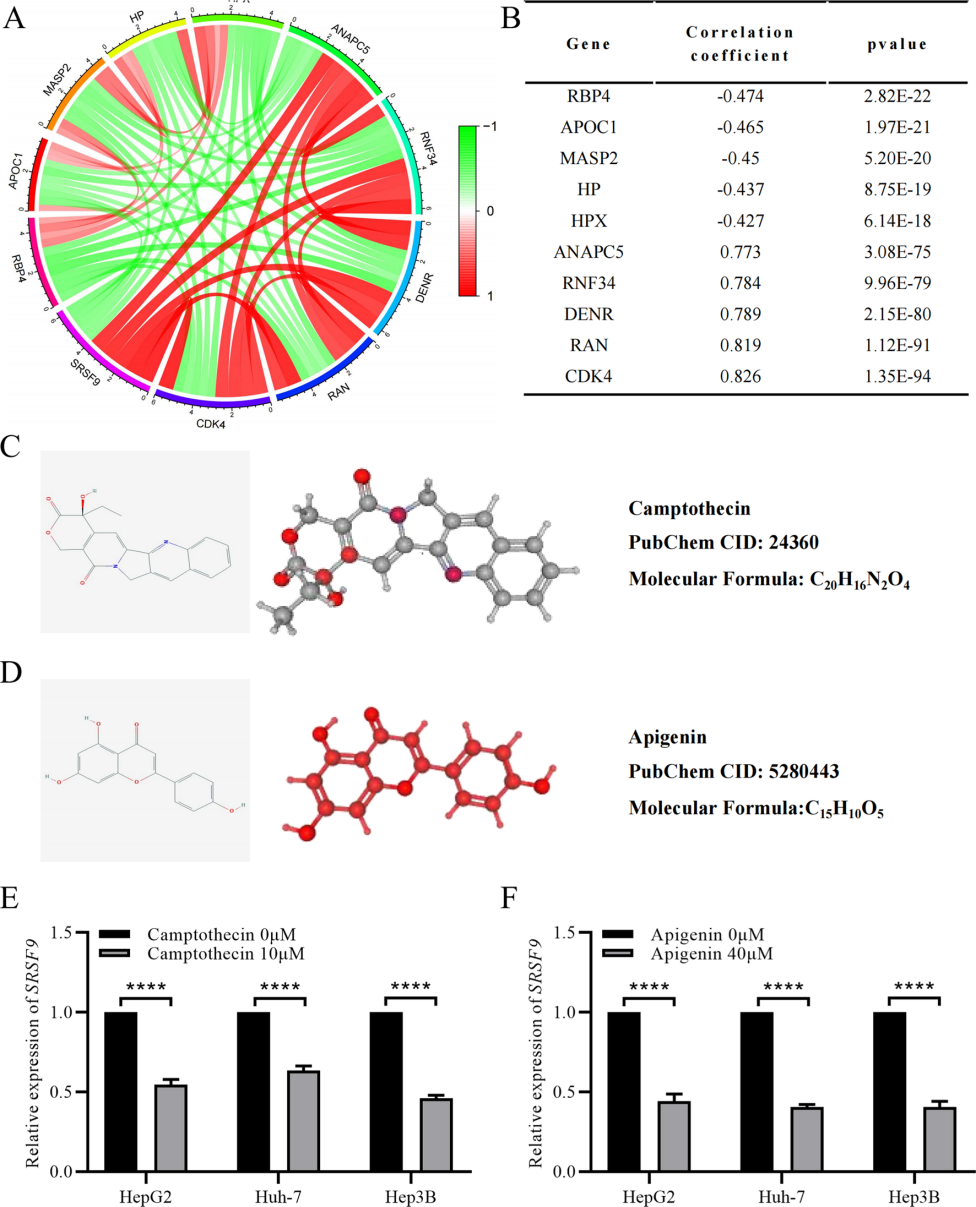
Co-expression analysis and CMap analysis of *SRSF9.* **A**, **B** Co-expressed genes with *SRSF9.* **C**, **D** Chemical formulae and 2D and 3D structures of candidate drugs, camptothecin and apigenin, from the PubChem database; **E**, **F** mRNA level of *SRSF9* in cells treated with camptothecin and apigenin. ****P < 0.0001 versus control group

A co-expression analysis was performed to explore the molecular mechanism underlying the effects of *SRSF9* in HCC. Hundreds of co-expressed genes were identified by setting a co-expression coefficient of less than -0.4 or greater than 0.4 and *P* < 0.05 as thresholds. The top five genes with the most highly positive (*CDK4*, *RAN*, *DENR*, *RNF34,* and *ANAPC5*) and negative (*RBP4*, *APOC1*, *MASP2*, *HP,* and *HPX*) correlations with *SRSF9* expression were selected as candidate co-expressed genes (Fig. 6A, B).

We regarded the genes with positive correlations as upregulated and those with negative correlations as downregulated. Using the CMap database, candidate small molecule drugs were obtained. We set the screening criteria as *P* < 0.001 and selected the first two small molecule drugs, camptothecin and apigenin, as likely to inhibit the expression of *SRSF9*, according to the maximum negative correlation coefficient. The chemical formula and 2D and 3D structures of candidate drugs were obtained from the PubChem database (Fig. 6C, D).

To verify whether the screened drugs have inhibitory effects on the expression of *SRSF9*, the hepatoblastoma cell line HepG2 and hepatocellular carcinoma cell lines Huh-7 and Hep3B were treated with camptothecin (10 μM) and apigenin (40 μM) for analyses of *SRSF9* expression by RT-PCR. As shown in Fig. 6E, F, the expression of *SRSF9* was significantly lower in cells treated with either drug than in the control group. These results suggest that camptothecin and apigenin could indeed reduce the expression level of *SRSF9* in HCC.

## Discussion

We detected a significant relationship between the occurrence of HCC and the dysregulation of *SRSF9* expression at the transcriptome level. The origin of HCC is accompanied by the activation of oncogenes and the inactivation of tumor suppressor genes [11]. Oncogenes are usually highly expressed in cancers [12], consistent with our results demonstrating that *SRSF9* expression is significantly increased in HCC the mRNA level based on TCGA data and at the protein level based on HPA data and laboratory HCC samples. Moreover, the activation of oncogenes can lead to the malignant progression of cancer, as the high expression of *SRSF1* in breast cancer is positively associated with a higher tumor grade [13]. Consistently, high *SRSF9* expression in this study was positively correlated with malignant physiopathological features of HCC. In addition, increased malignancy eventually leads to a poor prognosis [14]. For example, the increased expression of *YKT6* is correlated with the tumor size, Edmondson Grade, metastasis, and microvascular invasion and predicts a poorer prognosis in patients with HCC [15]. In this study, a Kaplan–Meier curve revealed that the increase in *SRSF9* expression can significantly reduce the overall survival time of patients. Further univariate and multivariate analyses suggested that *SRSF9* is an independent risk factor for prognosis in HCC. In general, these results suggest that *SRSF9* contributes to the pathogenesis of HCC and may influence disease progression.

DNA methylation in epigenetics is a very important gene regulatory mechanism, and low methylation in the genome generally contributes to the activation of oncogenes [16]. The hypomethylation status of the promoter region of *HJURP* is related to its high expression, which promotes the malignant progression of HCC and is associated with a poor patient prognosis [17]. Our results revealed that all seven methylation sites of *SRSF9* remained hypomethylated in HCC and showed identified that the methylation status of cg06116271 could negatively regulate the expression of *SRSF9*. Importantly, the hypermethylation of cg06116271 was associated with a better prognosis in patients with HCC. Therefore, the cg06116271 methylation site is a candidate biological target for the treatment of HCC.

Previous studies have suggested that DNA hypomethylation can activate the Wnt signaling pathway and promote tumor formation in HCC [18]. The activation of the Wnt pathway in HCC may be involved in the maintenance of tumor stem cells and cell proliferation, differentiation, infiltration, and migration [19]. In this study, we confirmed that *SRSF9* influences key proteins in the Wnt pathway by specifically knocking down *SRSF9* via in vitro experiments. As a key splicing factor, *SRSF9* affects the Wnt pathway and cell cycle pathway and influences the malignant progression of HCC by regulating cell proliferation and migration. Our comprehensive study of the role of *SRSF9* in the progression of HCC provides a precise and novel therapeutic target for diagnosis and treatment.

To promptly translate the study results for the benefit of patients with HCC, two drugs, camptothecin and apigenin, predicted to inhibit *SRSF9* expression were identified using the CMap database and verified using HCC cell lines. Numerous studies have reported the importance of camptothecin and apigenin for HCC therapy. Camptothecin downregulates the expression of Nrf2 and affects invasion, metastasis, and angiogenesis in HCC [20]. Apigenin can inhibit the proliferation of HCC by affecting the expression of microRNAs [21]. The results of this study further support the clinical value of camptothecin and apigenin, targeting *SRSF9*, providing ga basis for improving the rate of survival and prognosis of patients.

Although the combination of large-scale data analyses using public databases and in vitro experiments confirmed the impact of *SRSF9* on the prognosis of HCC from multiple perspectives, this study still had some shortcomings. First, according to a GSEA, *SRSF9* can regulate a variety of cancer-related cellular signaling pathways. However, it was not possible to verify the roles of all candidate cell signaling pathways. Second, as a retrospective analysis, all indexes with predictive value for prognosis should be included. Unfortunately, data for important clinical features, such as the Child–Pugh stage and aluminum and bilirubin levels, were lacking, which is an inherent disadvantage of public databases. These shortcomings will be addressed in future research.

## Conclusion

Our comprehensive analysis of the mechanism of action of *SRSF9* in HCC, high *SRSF9* expression regulated by the cg06116271 site predicted a poor prognosis. Furthermore, *SRSF9* can promote HCC proliferation and migration by regulating the Wnt pathway and cell cycle pathway. This study not only provides insight into the pathological mechanism underlying HCC but also provides a new target for diagnosis and treatment. Targeted therapy focusing on *SRSF9* may provide a personalized treatment strategy for patients with HCC, with better clinical effectiveness.

## Supplementary Information

**Additional file 1.** Figs. S1 to S3, Tables S1 to S2.

## Data Availability

The datasets generated and/or analysed during the current study are not publicly available due the data is being used in an ongoing, unpublished article, but are available from the corresponding author on reasonable request.
