# Supplementary material for: High expression of serine and arginine-rich splicing factor 9 (SRSF9) is associated with hepatocellular carcinoma progression and a poor prognosis
Source: BMC Med Genomics. 2022 Aug 15;15:180. doi: 10.1186/s12920-022-01316-7 (PMC9377132; doi:10.1186/s12920-022-01316-7)
Supplement: Supplementary file 1 — Additional file 1. Figs. S1 to S3, Tables S1 to S2. [file 12920_2022_1316_MOESM1_ESM.docx]

**
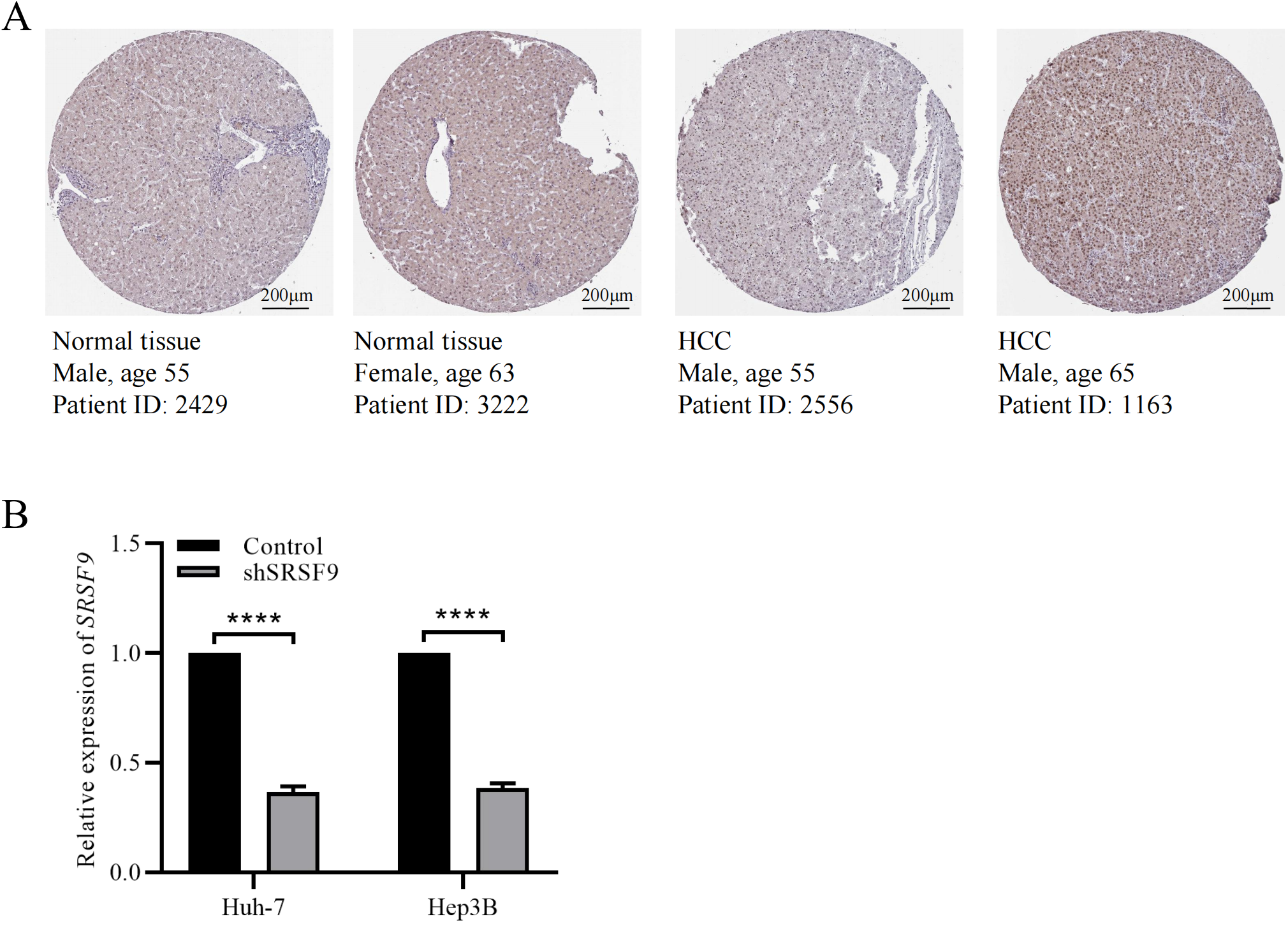
**

**Figure S1.** Expression of *SRSF9* in HCC tissues and cell lines. **A**, IHC results for *SRSF9* in HCC and normal tissues based on the HPA database; **B**, mRNA level of *SRSF9* in shRNA-treated HCC cell lines. *****P* < 0.0001 vs. control group.


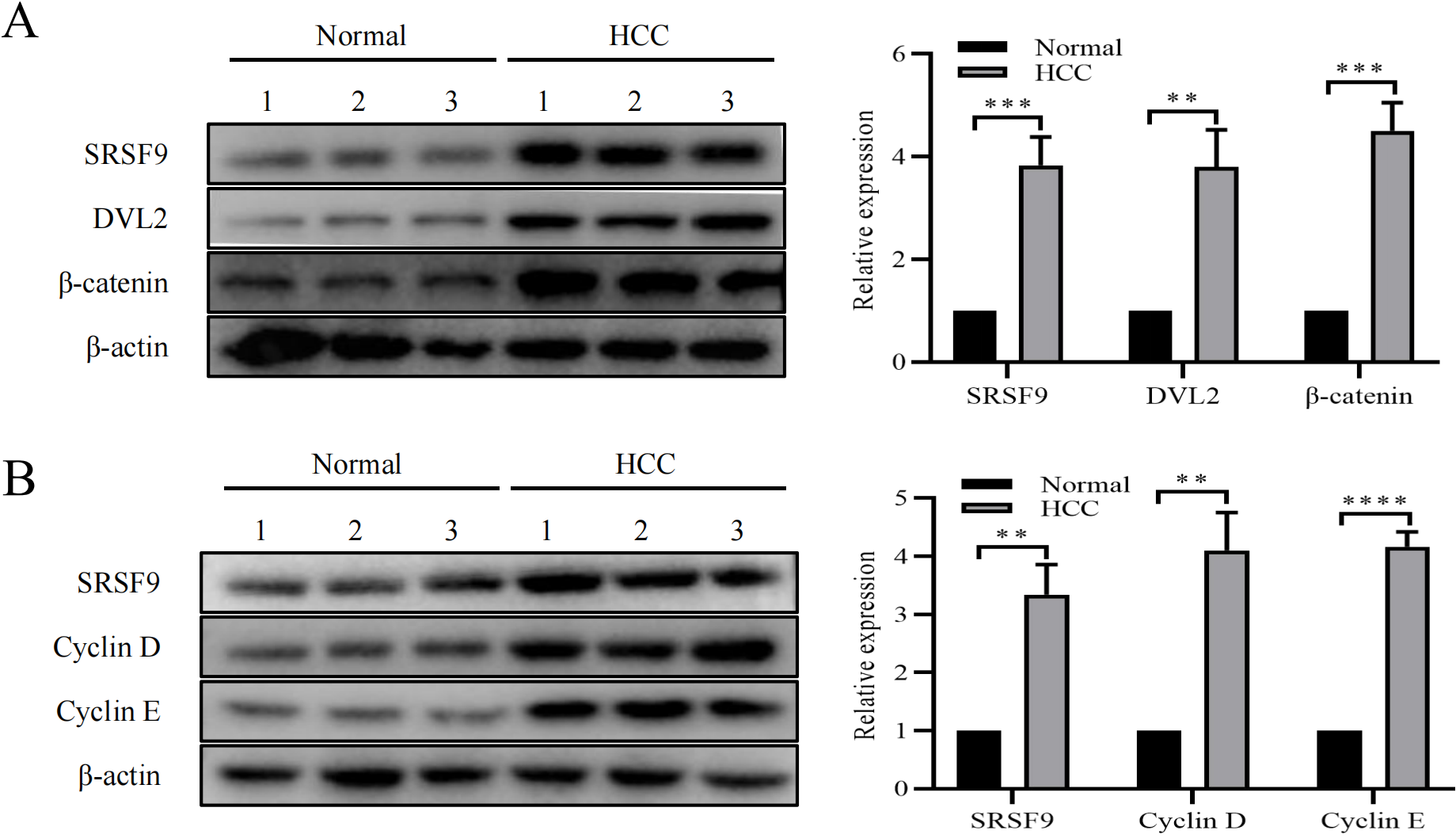


**Figure S2.** Expression of proteins in the Wnt signaling pathway and cell cycle pathway in HCC tissues. **A**, Expression of proteins in the Wnt signaling pathway in HCC and normal liver tissues from laboratory specimens; **B**, Expression of proteins in the cell cycle pathway in HCC and normal liver tissues from laboratory specimens. ***P* < 0.01, ****P* < 0.001, *****P* < 0.0001 vs. Normal group.

**Figure S3. Display of original western blots**

Hep3B

Huh-7

Huh-7

Hep3B

shNC

shSRSF9

shNC

shSRSF9

shNC

shSRSF9

shNC

shSRSF9


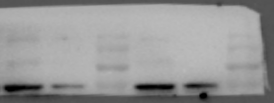

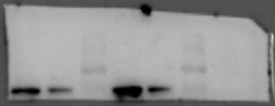


140kDa

140kDa

180kDa

180kDa

100kDa

DVL2 90-95 kDa

β-catenin 92 kDa

100kDa


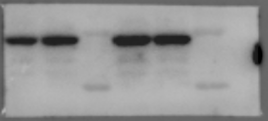


45kDa

β-actin 42kDa

35kDa


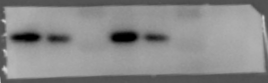


SRSF9 26kDa

25kDa

Normal

HCC

Normal

HCC

1

2

3

1

2

3

3

1

2

3

1

2


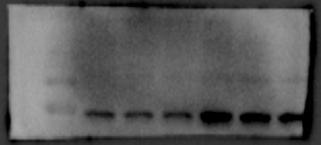

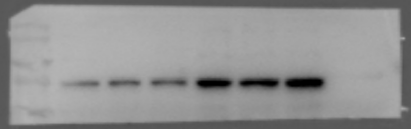


180kDa

180kDa

β-catenin 92 kDa

140kDa

100kDa

140kDa

100kDa

DVL2 90-95 kDa

70kDa


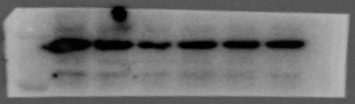


β-actin 42kDa

45kDa

35kDa


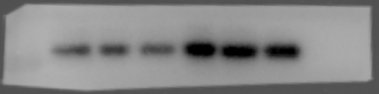


SRSF9 26kDa

25kDa

β-actin 42kDa

SRSF9 26kDa

25kDa

35kDa

45kDa

35kDa

60kDa

Cyclin D 34kD

Cyclin E 47kD

1

2

3

3

1

2

HCC

Normal

HCC

Normal

1

2

3

3

2

1

SRSF9 26kDa

β-actin 42kDa

shNC

shSRSF9

Huh-7

shNC

shSRSF9

Hep3B

45kDa

shNC

shSRSF9

Huh-7

shNC

shSRSF9

Hep3B

Cyclin D 34kD

Cyclin E 47kD

60kDa

45kDa

35kDa


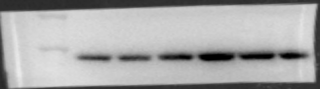

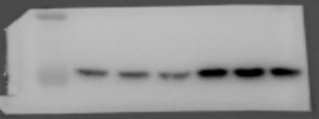

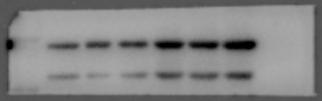

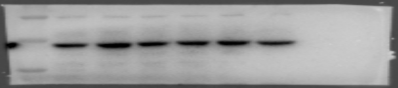

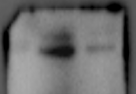

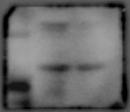

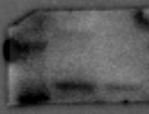

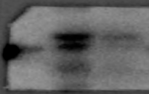

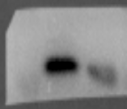

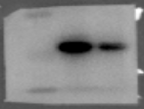

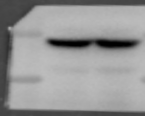

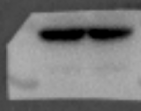


**Table S1 The detailed clinical features of LIHC patients in TCGA RNA-seq**

| **Covariates** | **Type** | **Total** | **Percentages(%)** |
| --- | --- | --- | --- |
| Age | <=60 | 171 | 47.9% |
|  | ＞60 | 186 | 52.1% |
| Histologic grade | G1 | 54 | 15.13% |
|  | G2 | 174 | 48.74% |
|  | G3 | 117 | 32.77% |
|  | G4 | 12 | 3.36% |
| Pathologic M | M0 | 259 | 72.55% |
|  | M1 | 3 | 0.84% |
|  | MX | 95 | 26.61% |
| Pathologic N | N0 | 246 | 68.91% |
|  | N1 | 4 | 1.12% |
|  | NX | 107 | 29.97% |
| Pathologic T | T1 | 179 | 50.14% |
|  | T2 | 87 | 24.37% |
|  | T3 | 78 | 21.85% |
|  | T4 | 13 | 3.64% |
| Family cancer history | NO | 202 | 56.58% |
|  | Unknown | 47 | 13.17% |
|  | YES | 108 | 30.25% |
| Gender | Female | 115 | 32.21% |
|  | Male | 242 | 67.79% |
| Race | Asian | 155 | 43.42% |
|  | Black or African American | 16 | 4.48% |
|  | Unknown | 9 | 2.52% |
|  | White | 177 | 49.58% |

**TABLE S2** | The gene set enriches the high *SRSF9* in TCGA RNA-seq database

| **GENE SET NAME** | **NES** | **NOM p-val** | **FDR q-val** |
| --- | --- | --- | --- |
| CELL CYCLE | 2.2537396 | 0 | 0.001078125 |
| SPLICEOSOME | 2.167767 | 0 | 0.00981272 |
| DNA REPLICATION | 1.99497 | 0.002074689 | 0.011618684 |
| WNT SIGNALING PATHWAY | 1.9338288 | 0 | 0.01600555 |

NES: normalized enrichment score; NOM: nominal; FDR: false discovery rate. Gene sets with NOM p-value <0.05 and FDR q-value <0.25 were considered as significantly enriched.
